# Overuse of Tuberculosis Surveillance Testing in Patients With Inflammatory Bowel Disease Compared to Non-IBD Patients on Biologic Therapy

**DOI:** 10.1093/crocol/otab026

**Published:** 2021-06-09

**Authors:** Sean Fine, Marc Vecchio, Joao Filipe Goncalves Monteiro, Eric Vecchio, Eric J Mao

**Affiliations:** 1 Department of Medicine, Division of Gastroenterology, Warren Alpert Medical School of Brown University, Providence, RI, USA; 2 Department of Medicine, Warren Alpert Medical School of Brown University, Providence, RI, USA; 3 Department of Gastroenterology and Hepatology at UConn Health, University of Connecticut School of Medicine, Farmington, CT, USA; 4 Department of Medicine, Division of Gastroenterology and Hepatology, University of California Davis School of Medicine, Sacramento, CA, USA

**Keywords:** inflammatory bowel disease, tuberculosis, interferon-gamma release assay, QuantiFERON-TB Gold, tuberculin skin test

## Abstract

**Background:**

Biologic treatment for moderate to severe inflammatory bowel disease (IBD) places patients at risk for infectious complications. Tuberculosis (TB) infection and reactivation can lead to serious morbidity and mortality for immunosuppressed patients. As a result, guidelines recommend screening for TB before starting biologic treatment, but a paucity of data remains on the utility of surveillance testing.

**Methods:**

We performed a retrospective chart review at a single academic center evaluating both IBD and non-IBD patients on biologic therapy. The primary outcome was to determine the number of subsequent surveillance tests performed after initial screening for latent TB in both patient groups.

**Results:**

A total of 188 patients (147 IBD and 41 non-IBD patients) on biologic therapy were included. Screening for TB before biologic treatment was performed in 56% of non-IBD patients versus 83% for patients with IBD (*P* = 0.0003). Of the total cohort, 65% had at least 2 follow-up surveillance tests for TB. Three or more surveillance tests were performed in 40% of patients with IBD versus only 13% for non-IBD patients (*P* = 0.0132). A total of 7 patients (4%) had an abnormal surveillance test. No patients were confirmed to have a diagnosis of TB or underwent treatment.

**Conclusions:**

Patients on biologic therapy unnecessarily undergo surveillance testing for TB. Patients with IBD on biologic therapy are screened annually for TB at a higher rate compared to non-IBD patients. Standardization of care among patients on biologic therapy is necessary to avoid excessive testing in areas with a low incidence of TB.

## Introduction

Inflammatory bowel diseases (IBD), Crohn disease (CD), and ulcerative colitis (UC) are chronic, progressive immune-mediated diseases of the gastrointestinal tract that lead to significant morbidity and mortality.^1^ Disease-related inflammation is the result of a dysregulated immune response causing a cascade of inflammatory cells and cytokines that have become the hallmark targets of therapies. Anti-tumor necrosis factor (anti-TNF) therapies remain an important part of the therapeutic armamentarium in the treatment of moderate to severe IBD.^2,3^ Newer biologic agents approved for CD and UC include Ustekinumab (IL12/23, Stelera) and Vedolizumab (α4β7, Entyvio), which specifically target alternate pathways of inflammation. Although biologic agents are able to regulate and suppress the dysregulated immune response seen in patients with IBD, they carry an increased risk for opportunistic infections.

Tuberculosis (TB) is a preventable communicable disease that is one of the leading causes of death worldwide according to the World Health Organization (WHO). Worldwide incidence rates of TB from 2015 to 2019 revealed an overall 9% decrease according to the 2020 WHO Global Tuberculosis Report.^4^ Geographic data from 2019 illustrate that nearly half of reported cases of TB cases were in regions of South-East Asia. India reported the highest number of cases at 2,640,000 followed by Indonesia, China, and the Philippines, 845,000, 833,000, and 599,000, respectively. Africa reported the second highest number of cases with Nigeria (440,000) and South Africa (360,000) reporting the most cases. Comparatively, data from the United States in 2019 demonstrated a TB case rate of 2.7 cases per 100,000 persons, the lowest levels on record. New York, Texas, California, and Florida represented 49.1% of all reported cases.^5^

Mycobacterium tuberculosis (MtB) infection often results in the development of latent infection (LTBI) with a 4%–6% lifetime risk of progressing to active MtB in immunocompetent hosts.^6^ However, the ability of the host to contain MtB is substantially reduced in patients who are immunocompromised. Immunosuppressed patients have a high likelihood of progression to active MtB, warranting routine screening.^6,7^ Biologic therapies, notably anti-TNF agents, have been associated with a 14-fold increase in TB reactivation compared to healthy controls due to impaired granuloma formation.^8,9^ Guidelines from gastrointestinal societies and the FDA (United States Food and Drug Administration) recommend screening for LTBI in all patients with IBD planning to initiate treatment with anti-TNF therapy, IL12/23 (Ustekinumab), or α4β7 (Vedolizumab).^10–13^

Screening for LTBI before initiation of biologic therapy in patients with IBD should include a detailed patient history, travel history, potential exposure risks, and laboratory testing. Testing modalities available to screen for LTBI include the traditional tuberculin skin testing (TST) and interferon-gamma release assays (IGRAs). QuantiFERON-TB Gold (QFT-gold) is one of the available IGRA tests that detects an immune response toward MtB proteins by measuring in vitro quantitative interferon-γ production from a patient’s collected peripheral-blood lymphocytes. QFT-gold results are reported as positive, negative, or indeterminate. An indeterminate is not interpretable for LTBI and occurs when patients exhibit a poor response to the TB mitogen secondary to T-cell dysfunction. IGRA testing has now become the standard screening tool for LTBI after recommendation by the Infectious Disease Society, American Thoracic Society, and Centers for Disease Control and Prevention (CDC).^6^ The advantages of IGRA over TST include single visit testing and minimized false-positive results; from either boosting due to repeated TST, previous Bacillus Calmette–Guerin vaccination, or infection with non-TB mycobacteria.^14^ There is also a higher degree of specificity with the IGRA test in low-endemic MtB regions.^15^ Although the IGRA test does have increased sensitivity and specificity compared to the TST in immunosuppressed patients, there remains a higher frequency of indeterminate results for this patient population.^16^ Indeterminate test results may lead to specialty consultations, additional testing, and delays in treatment.

With the trends over the years showing declining rates of TB, newer recommendations are being made in regard to surveillance screening for certain patient populations. Previous CDC 2005 guidelines for preventing MtB infection in health care settings included recommendations for baseline TB screening in all US health care personnel and annual testing for those working in settings with the potential for ongoing transmission.^17^ In 2019, new guidelines by the CDC encouraged no routine surveillance testing for TB in health care workers at any interval in the absence of a known exposure or ongoing transmission after initial baseline TB screening is performed.^18^ Patients with immune-mediated inflammatory diseases (IBD, rheumatoid arthritis, ankylosing spondylitis, psoriasis, uveitis, and multiple sclerosis) on biologic therapy remain a vulnerable population for LTBI. Despite the low incidence of TB cases in the United States, there are currently no definitive gastrointestinal societal guidelines for TB surveillance testing after an initial baseline screening test is performed. One of the possible driving forces responsible for TB testing practices may be infusion centers/hospitals that require evidence of a negative annual TB screen before renewing treatment plans. Furthermore, there appears to be variation in practice among specialists who care for patients on biologic therapy and the frequency at which TB surveillance testing is performed.

In the present study, the aim was to report the frequency of TB surveillance testing in a US cohort of IBD and non-IBD patients on biologic therapy and evaluate the utility of ongoing testing and its impact on clinical care.

## Materials and Methods

### Study Population and Variables

We conducted a retrospective descriptive study at Lifespan Hospital, the major teaching hospital associated with Brown Medical School (Providence, RI). Our database identified all patients (IBD and non-IBD) who were currently on biologic therapy between April 2015 and December 2016 and were being followed by Gastroenterology or other specialty practices within the institution (Rheumatology, Neurology, and Ophthalmology). Patients included in the study were aged 18 years or older who were started and continued on biologic therapy (infliximab (Remicade), adalimumab (Humira), certolizumab (Cimzia), golimumab (Simponi), natalizumab (Tysabri), vedolizumab (Entyvio), ustekinumab (Stelara), and etanercept (Enbrel).

For each of the above-identified patients on therapy, the subject’s information (demographics, disease type), biologic start date time, prebiologic TB screening results, and all subsequent TB surveillance tests performed after the initial baseline were extracted retrospectively. Follow-up testing for TB was identified in the chart as either TST or QFT-gold testing. A positive TST test was identified with a skin induration of more than 5 mm and a QFT-gold test result was either reported as positive, indeterminate, or negative by our laboratory. Charts were reviewed to assess for subsequent testing for TB in each patient. Study data were collected and managed using REDCap, an encrypted electronic data capture tool hosted by Lifespan.

### Statistical Analysis

The analysis was conducted in SAS software, where chi-square and Student *t* tests were performed for descriptive analyses to report demographics, type of medication, screening test result, screening method before biologic treatment, and the number of subsequent tests for TB.

## Results

One hundred and eighty-eight patients on biologic therapy were included and analyzed in 2 groups (Table 1): 147 (78.2%) patients in the IBD group (UC and CD) and 41 (21.8%) patients in the non-IBD group (Table 2). Patients with IBD were younger (39.9 years) compared with the non-IBD patients (51 years), *P* < 0.0001. One hundred and thirty-one patients (69%) were female and the predominant patient race was white or Caucasian (82%). Anti-TNF-α therapy accounted for 90% (169) of biologic use in both the IBD and non-IBD groups (Table 1).

**Table 1.** Characteristics of non-IBD and IBD patients on biologic therapy

Patient characteristics			
	Non-IBD (n = 41)	IBD (n = 147)	Overall (n = 188)
Age, mean (range)****	51.0 (22–74)	39.9 (18–75)	42.3 (18–75)
Male gender, n (%)	8 (19.5)	49 (33.3)	57 (30.3)
White or Caucasian race, n (%)	30 (73.2)	124 (84.4)	154 (81.9)
Diagnosis, n (%)			
Ulcerative colitis	—	31 (21.1)	31 (16.5)
Crohn disease	—	115 (78.2)	115 (61.2)
Medication type			
Anti-TNF	34 (88.9)	135 (91.8)	169 (89.9)
Natalizumab	2 (4.9)	0 (0.0)	2 (1.1)
Vedolizumab	0 (0.0)	9 (6.12)	9 (4.8)
Ustekinumab	1 (2.4)	2 (1.4)	3 (1.6)
Etanercept	5 (12.2)	0 (0.0)	5 (2.7)

*P < 0.05; **P < 0.01; ***P < 0.001; ****P < 0.0001.

IBD, inflammatory bowel disease; TNF, tumor necrosis factor.

**Table 2.** Characteristics of non-IBD patients

Disease type	Number (%)
Retinitis	1 (2.4)
Uveitis	1 (2.4)
Psoriatic arthritis	8 (19.5)
Rheumatoid arthritis	15 (36.6)
Psoriasis	4 (9.76)
Ankylosing spondylitis	5 (12.2)
Juvenile rheumatoid arthritis	1 (2.4)
Multiple sclerosis	2 (4.8)
Behcet disease	1 (2.4)
Seronegative spondyloarthritis	3 (7.3)

Overall, 77% (145) of patients were found to have had a documented screening test for TB before initiating biologic therapy (Table 3). In the IBD group, 83% (122) of patients had a documented TB test performed before initiating biologic therapy compared to only 56% (23) in the non-IBD patients (*P* = 0.0003). Of the patients in the non-IBD group that had reported screening, QFT-gold testing was performed the majority of the time versus TST (74% vs 26%, respectively). Screening for TB in the IBD group had a similar distribution between the use of TST (53%) and QFT-gold (47%). The overall rate of a positive screening TB test for the cohort before biologic therapy was low at 1.6% (3 patients total). In the IBD group, 1 patient of the 145 (0.7%) screened positive for TB.

**Table 3.** Screening rates for tuberculosis before biologic therapy of non-IBD and IBD patients

	Non-IBD (n = 41)	IBD (n = 147)	Overall (n = 188)
Documented screening before treatment***	23 (56.1)	122 (83.0)	145 (77.1)
Screening type before treatment, n (%)			
TST	6 (26.1)	65 (53.3)	71 (48.6)
QFT-gold	17 (73.9)	57 (46.7)	74 (50.7)
Initial screening result, n (%)			
Positive	2 (4.9)	1 (0.7)	3 (1.6)
Negative	21 (51.2)	121 (82.3)	142 (75.5)

*P < 0.05; **P < 0.01; ***P < 0.001; ****P < 0.0001.

IBD, inflammatory bowel disease; TST, tuberculin skin test; QFT-gold, QuantiFERON-TB gold.

**Figure 1.**
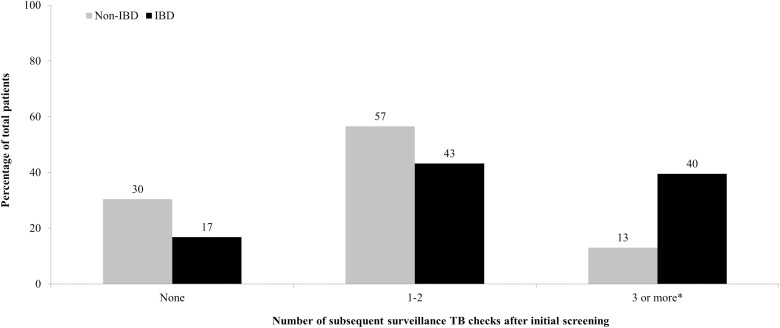
A number of subsequent surveillance TB checks after initial screening on non-IBD and IBD patients after starting biologic therapy. IBD, inflammatory bowel disease; TB, tuberculosis. *P < 0.05.

Of the 145 patients who had TB testing before biologic therapy, 142 (97%) had continued documentation in electronic medical records of ongoing follow-up with providers. In the non-IBD group, 67% (14/21) of patients had at least one follow-up surveillance test performed, whereas 84% (102/121) of patients in the IBD group had at least one test performed. However, this was not statistically significant (*P* = 0.0680, Table 4). When the number of subsequent testing was broken down into different groups, there was no difference between the non-IBD and IBD groups when 1–2 follow-up surveillance tests were ordered. However, having 3 or more surveillance tests performed after the initial screening was seen significantly more often in the IBD versus the non-IBD group (40% vs 13%, *P* = 0.0132, Figure 1).

**Table 4.** Abnormal surveillance testing for tuberculosis of non-IBD and IBD patients on biologic therapy

	Non-IBD (n = 41)	IBD (n = 147)	Overall (n = 188)
Had at least 1 follow-up surveillance test	14 (67.7)	102 (84.3)	116 (81.7)
Results of TB test during surveillance, n (%)			
Abnormal	2 (4.9)	5 (3.4)	7 (3.7)
Negative	28 (68.3)	126 (85.7)	154 (81.9)

IBD, inflammatory bowel disease; TB, tuberculosis.

There were only 7 (3.7%) abnormal tests during surveillance follow-up, 2 in the non-IBD group and 5 in the IBD group. All the abnormal tests were QFT-gold, with 5 of 7 found to be indeterminate, 1 of 7 felt to be false positive, and 1 of 7 positive (Table 5). Documentation of follow-up testing was performed in 5 of 7 patients with either repeat QFT-gold or TST. The results of the testing were negative. Of the 7 patients with abnormal testing, 70% were females and 5 of 7 were below the age of 40. There were no identifiable TB risk factors documented for patients and occupations included a kitchen worker, 2 students, pharmacist, Vice President of a company, and 2 patients who were retired. Two of the patients resided in the same city of Pawtucket in Rhode Island, otherwise, the remainder of the patients were spread out around the state in different zip codes. The patient with the QFT-gold positive test (rheumatoid arthritis) had previously been treated for TB and had a history of previously residing in Guatemala. In response to the abnormal TB test result, the patient did not have any documented signs or symptoms concerning for re-infection and no further intervention was performed. Of all the patients with an abnormal surveillance test, 1 of 7 (0.5%) had a documented delay in biologic therapy.

**Table 5.** Patients with abnormal. TB Surveillance Testing

Non-IBD	IBD	Surveillance Test	Result Type	Follow-up Test	Documented Treatment of TB	Delay in Treatment
Psoriasis	—	QFT-gold	Indeterminate	None	None	No
—	Crohns	QFT-gold	Indeterminate	QFT-gold Negative	None	No
—	Crohns	QFT-gold	Indeterminate	TST Negative	None	No
—	Crohns	QFT-gold	Indeterminate	TST Negative	None	No
—	UC	QFT-gold	Indeterminate	TST Negative	None	No
—	Crohns	QFT-gold	False Positive	None	None	Yes
Rheumatoid Arthritis	—	QFT-gold	Positive	QFT-gold Negative	None	No

IBD, inflammatory bowel disease; TB, tuberculosis; QFT-gold, QuantiFERON-TB gold; TST, tuberculin skin test; UC, ulcerative colitis.

## Discussion

The use of biologic therapy for the management of immune-mediated inflammatory diseases has been instrumental in preventing disease progression; however, the inherent potential infectious complications continue to impact clinical care practices. In this study, we found patients with IBD on biologic therapy had higher rates of surveillance testing for TB than non-IBD patients on biologic therapy. The surveillance testing abnormality rate was low at 3.7%, with no cases of true seroconversion leading to any treatment and no meaningful changes or alteration of care as a consequence of testing results except from one patient having a delay in treatment. Documentation of prebiologic TB testing was found at a higher rate in patients with IBD compared to non-IBD patients, 83% versus 56%, respectively.

Reactivation of latent TB is a potential infectious complication that may arise with biologic therapy, particularly agents targeting anti-TNF-α. Cases of latent TB infection became evident early on in clinical use with infliximab. In 2001, an adverse event reporting system through the FDA found 70 cases of TB reactivation at a median of 12 weeks after therapy initiation, with 68% of those patients developing an infection after 3 or fewer infusions.^19^ After these findings, a warning was added to the approved labeling for infliximab detailing the potential risk for TB. Raval et al^20^ suggested the risk of TB reactivation with Infliximab use may not be generalizable to the entire treated population and suggested possible risk factors to consider in high-risk patients. These risk factors included the use of concomitant immunosuppressants, being born into or having spent extensive time (at least 3 months) in an area where TB is endemic, or a history of latent or active TB. Based on this potential risk, screening for TB should be performed before starting biologic therapy in all patients and should entail a combination of the patient’s history, physical exam, and testing (TST or IGRA).^3,21^ In this study, we found Gastroenterologists (GIs) caring for patients on biologics had a higher rate of completing this process compared to non-GI providers. Although documented adherence was not 100%, meeting this benchmark was important to note given the possible association and the fact 90% of the patients in our cohort were on anti-TNF-α therapy.

There are currently no recommendations from gastroenterology societies that define any reasoning or need to perform surveillance TB testing. A significant proportion of patients in this study with IBD underwent multiple (3 or more) follow-up surveillance tests compared to non-IBD patients (*P* = 0.0132) with no true seroconversions and no patients requiring treatment for Mtb. The reasoning behind this heightened testing practice may stem from a lack of awareness compared to colleagues in other specialties utilizing biologics where updated practice guidelines provide more recommendations. The American College of Gastroenterology guidelines on Preventative Care in Inflammatory Bowel Disease did not address the topic of TB testing and monitoring.^22^ The Crohn’s and Colitis Foundation Health Maintenance checklist recommends that all IBD patients be screened for latent TB at baseline and an annual risk assessment for TB be performed in all patients, but no specific recommendations are made for IBD patients on biologic therapy.^23^ The American College of Rheumatology 2012 guidelines for patients with Rheumatoid Arthritis state patients treated with biologic agents should undergo annual testing if TB exposure is likely, but does not endorse yearly surveillance in all patients.^24^ The Joint American Academy of Dermatology and National Psoriasis Foundation 2019 guidelines state yearly testing for latent TB should be performed in high-risk patients on biologics, but determining which low-risk patients to test yearly should be done at the discretion of the treating provider.^25^ More recently, studies to assess the utility of surveillance testing for TB by rheumatologists have demonstrated this practice to be costly and of low clinical value. Pattanaik et al^26^ conducted a retrospective study of rheumatology patients at the Tennessee, VA and looked at the rate of surveillance testing. A total of 420 tests were performed on the 123 patients included in the study and patients were screened on average every 1.2 years for 4.3–12 years. Only 1 out of 123 patients (0.8%) seroconverted for TB. Similarly, Khanna et al^27^ retrospectively evaluated 5212 patients on biologic therapy with at least 1 repeat testing and found the majority of tests to be negative (87%). Only one case of active TB was found in the entire study cohort (0.01%) and this was in a patient who had a significant risk factor. The authors concluded that surveillance testing was of little value and the decision to test should be determined after identifying patients who have high-exposure risk factors for TB. One small retrospective descriptive study of 44 patients with IBD on anti-TNF treatment looked at the rate of seroconversion after an initial baseline negative test. Abitbol et al^28^ found that even after an initial negative screen, the risk for conversion occurred in 57% (25/44) of patients. However, over half of the patients with positive follow-up tests had an occupational work exposure or traveled to endemic countries. Another study in patients with IBD looked at the prevalence of TB seroconversion for patients on anti-TNF therapy and again found rates to be very low. Hou et al^29^ demonstrated a TB reactivation rate of 2.8 per 10,000 patient-years of exposure to anti-TNF therapy. The 2 patients with reactivation had previously documented treatment for TB before starting therapy. Similarly, in our patient cohort, we found no cases of seroconversion or reactivation of TB in the 188 patients who were treated with biologic therapy.

The variation of surveillance screening practices for TB in patients on biologic therapy may stem from several different issues. In the United States, where TB rates now seem to be at all time low, 4 areas account for half of the documented infections: California, Texas, New York, and Florida.^5^ Providers caring for patients who reside in these areas may feel that the benefits outweigh the risk of yearly surveillance and can easily be accomplished through blood testing with the available IGRA. A study evaluated a cohort of patients with rheumatoid arthritis on anti-TNF therapy in Southern California and the rate of seroconversion to a positive TB test.^30^ Goel et al found the conversion rate to be elevated at 9.4% at a median time of 31 months on therapy and there were no known risk factors, though Hispanics were at a higher risk (75%) compared to non-Hispanic whites (25%). Notably, patients in this study only had QFT-gold tests performed and TST was not utilized. The authors concluded that annual surveillance for LTBI should be strongly considered for their population given the local prevalence. However, patients who do not reside in these 4 states and without risk factors have little to no benefit of having repeat testing. As was demonstrated in our cohort, despite the high numbers of repeat surveillance testing, there were no cases of seroconversion. Rhode Island in 2019 had just 14 cases of TB, this was down 30% from 2018, with 93% of the cases in foreign-born patients.^31^

Infusion centers and agencies may also represent another possible source for the variation in TB surveillance testing. The previous recommendations by societies calling for annual testing and drug labels^32^ have likely impacted practice protocols that have been implemented in these care settings. Another potential driver in the requirement or recommendation of surveillance testing may stem from insurance mandates.^33^ Specialists with awareness to societal guidance and recommendations on TB surveillance testing may be more likely not to perform and decline testing for patients even if being recommended/required by centers, agencies, or insurances. Our study may reflect this disparity in practice as providers caring for patients with IBD were more likely to order a higher number of surveillance tests compared to those treating non-IBD patients.

It has been illustrated that patients with IBD incur a 3-fold higher direct cost of care compared to non-IBD controls.^34^ With the number of patients being diagnosed and treated with biologic therapy in the United States continuing to rise, efforts should focus on interventions to reduce costs in the health care system. Eliminating testing would be a way to lessen the burden of increased health care expenditures over time. The estimated cost of an IGRA test for TB can cost around $50.^35,36^ Although this number may not seem significant, the interval repetition will be a factor over time. Khanna et al^27^ found 5212 patients at Cleveland Clinic had repeat TB surveillance testing with an additional 9611 tests performed resulting in an estimated expenditure of $1,201,375. Patients in this cohort accumulated $136,200 in costs as a result of additional testing for an indeterminate QFT-gold with none leading to any diagnosis or treatment for LTBI.

Our study has limitations due to the retrospective nature of the data collection. Although the majority of TB testing appeared to be performed for routine annual surveillance, there may have been other reasons behind testing such as switching to new treatments, patient cessation of treatment, or the restarting of treatment. However, these instances would still continue to support the notion that repeat testing is unnecessary if patients had an initial baseline negative screen and did not have any new risk factors for TB. It is also important to consider that despite the low rates of seroconversion we observed, there still may be pockets of areas in low-incidence places with a risk of TB exposure that providers should be aware of when treating patients on biologic therapy. Our study continues to add to the limited literature available on TB seroconversion for low-risk patients on biologics. Finally, the difference in the ordering rate of TB testing among specialty practices caring for patients on biologic therapy was highlighted in our study. Gastroenterology providers were more likely to order higher numbers of repeat testing compared to other specialties. Identifying the potential reasons behind this difference, such as lack of guidelines, may play a pivotal role in narrowing the gap to ensure a standard of care to avoid unnecessary testing and reduce healthcare costs.

## Conclusions

We recommend that patients with IBD on biologic therapy in low-incidence regions, such as the majority of the United States, should not undergo yearly testing for TB unless they are considered a high-risk group or have a potential exposure. It is important for physicians to be aware of the regional prevalence of TB in conjunction with history taking on an annual basis to determine if any additional surveillance testing for TB is required (Table 6). Rationale for continued surveillance TB testing should be documented in the electronic medical record.

**Table 6.** Factors providers should consider surveillance TB testing for patients on biologic therapy

*Do you practice in a region with a high or low incidence of TB?* • If low and NO high-risk factors, then avoid annual surveillance testing • If low AND any high-risk factor present, then perform annual surveillance testing • If high, then perform annual surveillance testing	
	High-risk factors*
	• HIV infection • IV drug use • Homeless • Close contact with a person infected with TB • Residents and employees of high-risk congregate settings (homeless shelters, correctional facilities, nursing homes, and resident homes for patients with HIV) • Health care workers who serve high-risk patients

*Center for Disease Control and Prevention.^37^

TB, tuberculosis.

## Data Availability

Data were not publicly available.
